# Automated detection of koalas using low-level aerial surveillance and machine learning

**DOI:** 10.1038/s41598-019-39917-5

**Published:** 2019-03-01

**Authors:** Evangeline Corcoran, Simon Denman, Jon Hanger, Bree Wilson, Grant Hamilton

**Affiliations:** 1 School of Earth, Environmental and Biological Sciences, Queensland University of Technology, 2 George Street, Brisbane, QLD 4000 Australia; 2 School of Electrical Engineering and Computer Science, Queensland University of Technology, 2 George Street, Brisbane, QLD 4000 Australia; 3 Endeavour Veterinary Ecology Pty Ltd, 1695 Pumicestone Rd, Toorbul, QLD 4510 Australia

## Abstract

Effective wildlife management relies on the accurate and precise detection of individual animals. These can be challenging data to collect for many cryptic species, particularly those that live in complex structural environments. This study introduces a new automated method for detection using published object detection algorithms to detect their heat signatures in RPAS-derived thermal imaging. As an initial case study we used this new approach to detect koalas (*Phascolarctus cinereus*), and validated the approach using ground surveys of tracked radio-collared koalas in Petrie, Queensland. The automated method yielded a higher probability of detection (68–100%), higher precision (43–71%), lower root mean square error (RMSE), and lower mean absolute error (MAE) than manual assessment of the RPAS-derived thermal imagery in a comparable amount of time. This new approach allows for more reliable, less invasive detection of koalas in their natural habitat. This new detection methodology has great potential to inform and improve management decisions for threatened species, and other difficult to survey species.

## Introduction

Detecting individual animals is key to monitoring the presence and distribution of threatened and invasive species^1,2^. Traditional surveys using direct observation are subject to observer bias, often require a large survey effort, and may be hindered by terrain that is difficult to navigate or access^3–8^. Aerial survey technologies such as Remotely Piloted Aircraft Systems (RPAS), otherwise known as UAVs or drones, are increasingly being used to collect image data of wildlife populations, with the potential to reduce detection error, overcome problems of site inaccessibility, and gain more detailed information regarding population size and trends of surveyed species^9–12^.

Image data collected from aerial surveys are often interpreted manually, but this can be tedious, time-consuming, and is still subject to interpreter bias with a tendency towards low probability of detection and high rates of false negative error^13,14^. Automated detection of individual animals in remotely sensed imagery can reduce bias and increase accuracy and precision of wildlife surveys, but few methods have been developed and tested in the field^13,15,16^. For mammals, the automated detection methods shown to be most accurate thus far have been applied to thermal imagery, as the large temperature gradient between mammals and their background environment allows computer vision to easily detect and count their thermal signatures^2,17^.

The monitoring and management of koalas (*Phascolarctus cinereus*), an Australian mammal species of conservation concern, has the potential to benefit greatly from development of a robust automated detection method using RPAS and thermal imagery^18,19^. Koala populations are often widely and unevenly distributed and frequently occur on private property, making them difficult and time-consuming to survey accurately by direct observation^18,20^. They are also cryptic in nature and inhabit environments with complex canopy cover, which significantly lowers the probability of detecting all individuals through direct observation both on the ground and in colour photographic imaging^21^. Experienced observers have been found to detect only 60–75% of koalas present within a survey area, even with relatively sparse and unobstructive vegetation^21^. A recent study in south-east Queensland demonstrated that experienced koala spotters were able to observe approximately 70% of koalas present^22^. The probability of detection has also been shown to decline significantly with decreased expertise and increased vegetation density, with non-expert observers found to observe only 23% of koalas present in an area^21,22^.

Previous attempts to automatically identify wildlife including nesting seabirds, livestock, bears and large African mammals using RPAS and other aerial imaging have exhibited high probability of detection of 43–100%, however these studies were conducted on small sample sizes, high-density populations, or a combination of both^23–29^. Koalas are also found in more complex habitats than have been surveyed in the majority of prior automated wildlife detection studies, which presents an added challenge^22^. Attempts to automatically detect large species such as deer in semi-natural forest habitats have given a high probability of detection (75–100%)^30,31^, but we have been unable to find any other published studies using automated detection methods for wildlife in RPAS-derived thermal imaging in complex environments. It therefore remains an open challenge to determine the accuracy and precision of such methods for detecting low-density cryptic species in a natural environment with complex canopy structure.

Determining the probability of detection for any species using a new methodology in a natural setting remains crucial to formulating effective management strategies^1,19^. Little is known, however, about the accuracy and precision of automated detection in RPAS-derived thermal imagery compared to manual assessment of the survey footage for wildlife in complex environments^1,18^. The aim of this study was to assess the accuracy and precision of a new automated detection method for koalas in RPAS-derived thermal imaging using published object detection algorithms in a realistic field setting with complex vegetation. Automatic and manual detections of koalas in RPAS thermal imaging from surveys conducted in Petrie, Queensland were matched with locations of a large population of radio collared koalas to verify the accuracy and precision of this novel detection method.

## Materials and Methods

### Algorithm Description and Workflow

**Figure 1:**
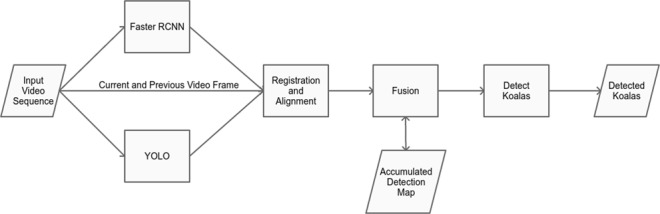
Flowchart of the koala detection pipeline. An input video was processed by DCNN detectors (Faster RCNN and YOLO), and these frames were aligned to a common coordinate frame through registration. The outputs of both methods were combined with previous detection results, and areas that record detections consistently over a number of frames were detected as koalas.

Although deep learning-based detection techniques are typically trained and deployed on data captured at ground level, a number of existing techniques have been applied to detect objects from aerial imagery, achieving promising results^32,33^. In this study, two of these deep convolutional neural network (DCNN) object detection methods that are well-established and popular were used to detect koalas: Faster-RCNN^34^ and YOLO^35^; and their outputs combined in a pipeline depicted in Fig. 1. No architectural changes were made to either network.

The first step in this process involved detecting koalas in RPAS-derived thermal image frames separately with each DCNN (Faster RCNN and YOLO). This was achieved by giving each DCNN an input image, which then produced a list of bounding boxes, a label that indicated what type of object was within the box, and a confidence value between 0 and 1 that denoted how confident the detector was that the detection was correct, with 1 being very confident^35,36^. Object detections were then filtered by confidence, excluding all detections below a 0.05 confidence level. In this case, the detectors were only trained to recognise koalas, and so only a single label was used; however, if multiple labels were used the approach could be trivially extended to multiple species.

The second step of the algorithmic process was transforming the list of bounding boxes for koala detections in single frames from each DCNN into ‘heat maps’. These heat maps were images the same size as the input with any pixel within one of the detected bounding boxes being set to 1 and all other pixels were set to 0, resulting in a binary image that indicated where detections were. For each frame a heat map was created for both Faster RCNN and YOLO.

The final step involved combining these two detectors by averaging these heat maps, allowing regions that are consistently detected across multiple frames to be identified. This improved the robustness of the automated detection method by allowing problems such as noise or occlusions that would prevent a koala from being detected in a single frame to be overcome by some extent. To average heat maps the images were first aligned or ‘registered’. As the RPAS was continuously moving, images from consecutive frames were offset. Key-point detection and matching was therefore used to estimate a homography, or match between pixels that had moved from between consecutive images. In particular, the ORB detector^36^ was used to compute a set of point matches between images captured at time t and at time t-1. If sufficient point matches were found (4 are required as a minimum) then a homography was computed between the two images. The feature extraction, matching and homography computation were all computed using OpenCV^37^. The resultant homography was then used to transform the averaged heat map from frame t-1 to the coordinate space of frame t. The average was then updated as follows:

$$A(t)=\frac{I(t)}{N}+\frac{N-1}{N}A(t-1),$$

Where A(t) is the accumulated image at time t, I(t) is the heat map computed from the detectors at time t, and N is a constant that controls how fast the accumulator is updated. This allows for a simple moving average of the detections to be kept. If a homography could not be found between frame t and t-1, the accumulated heat map was reset, such that A(t) = I(t).

**Figure 2:**
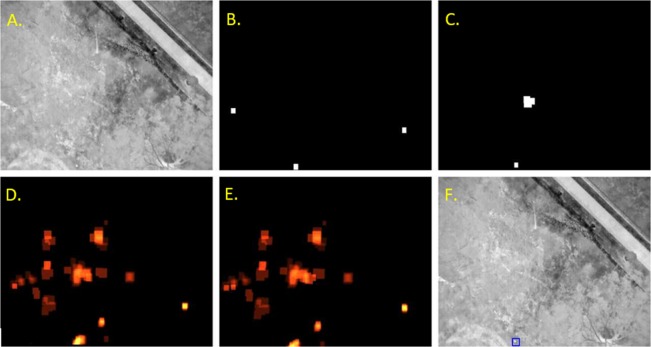
Example detection maps for a single image, and the combined results. (**A**) Input image, (**B**) Heat map for the F-RCNN, (**C**) Heat map for YOLO detectors, (**D**) the previous accumulator image, (**E**) updated accumulator incorporating the new heat map, and (**F**) the detected koala in the original image.

Candidate ‘koala’ signatures were then detected from the averaged heat map as shown in Fig. 2. Any region that was consistently detected over a number of frames was identified by applying the 0.05 confidence threshold to the heat map. Following this, connected component analysis was used to locate continuous regions, and if these regions had a height and width between 3 and 30 pixels they were accepted as candidate signatures.

To reduce repeated detections of the same koala, detected koalas were tracked over frames using a simple greedy tracking algorithm^38^. The result of this overall process was a set of koala detections that spanned multiple frames, which could be exported by the algorithm for manual review.

### Algorithm Training

Although DCNNs typically require thousands or even millions of observations to train, a technique commonly referred to as fine-tuning allows this training cost to be greatly reduced^32,33,38^. Fine-tuning takes a network trained on a very large, general purpose dataset and adapts the model to new task by adapting the learnt weights on a small image dataset^32,33,38^.

To fine-tune the algorithm used in this study a small corpus of thermal RPAS koala images was annotated. For these images, koalas were detected using a combination of in-field observations guided by radio-tracking; and unaided manual inspection where no radio-collar data was available. The model training was conducted in an iterative process as follows:

1. An initial model, consisting only of positive training examples (images containing koalas) was created using previously captured data from South East Queensland. This corpus was approximately 500 images in size. This initial data was accessed from previous flights conducted in the areas of Coomera and Pimpana, South East Queensland, Australia in 2016. It was captured using similar cameras and flight profiles.
2. The initial model was used to detect koalas in newly captured data from Petrie, Queensland and results were analysed for errors. Images of koalas not detected with the automated approach were added to the training set, and false detections (typically people, kangaroos, and vehicles) were added to the training data as negative examples. This data was then used to re-train the model, starting from the original image-net weights (i.e. the fine tuning process was repeated).
3. Step 2 was then repeated as more data was captured, to further improve the model. This allowed gradual capturing of more data and introduced more variation into the dataset, leading to a more robust model.

Faster RCNN was fine-tuned for 70,000 steps using weights trained on ImageNet data, and fine-tuned with default learning parameters and a batch size of 32. Both the VGG16 and ResNet101 architectures were investigated and found to offer similar performance^39,40^. For YOLO, weights trained with MS-COCO were used and fine-tuned with default learning parameters and 1000 epochs with a batch size of 32.

The total training dataset contained 918 images, and 732 annotated koalas. This dataset contained images captured from the Petrie test site on five days: the 27^th^ February 2018, 22^nd^ and 23^rd^ of May 2018, and the 12^th^ and 13^th^ of June 2018, which correspond to surveys North 1–3, South 1–2 datasets. As such, in the evaluation we present results for North 1–3 and South 1–2 separately to the rest of the collected data. It should however be noted that only a small subset of frames from these datasets were used for training.

The 918 images used represents significantly more images, and a similar number of overall detections as used in previous work^38^. Large numbers of negative examples were deliberately avoided to ensure that the model has maximum probability of detection. Instead this approach relies on the fusion of multiple detectors and spatiotemporal accumulation of detection results to help suppress false detections.

### Survey Site Selection and Data Collection

Two eucalypt forest survey sites in north and south Petrie Mill, Queensland were selected for this study. These sites were selected because they contain a population of 48 koalas that had previously undergone extensive tracking and radio collaring by field ecologists^41^. The north site was bounded by high-traffic roads and urban areas and the south site was bordered by a river significantly impeding koala immigration or emigration from the north or south sites, leading to a low estimated immigration rate^22^. The koalas isolated within the survey sites were tracked and surveyed by a ground team on the same day RPAS missions were conducted, and their GPS locations recorded to enable validation of automated detections.

A total of eleven RPAS surveys were conducted between February and August 2018 with six missions flown at the North site, three to gather training and testing datasets each, and five missions at the South site, two for gathering training data and three for collecting testing data (Table 1). The missions were carried out using a Matrice 600 Pro and A3 flight controller (DJI, Shenzhen, China) with a FLIR Tau 2 640 thermal camera (FLIR, Wilsonville, Oregon, United States of America) mounted to the underside with a gyro-stabilized gimbal to reduce vibration within frames. The thermal camera had a resolution of 640 × 512 pixels, 13 mm focal length lens and 9 Hz frame rate. The RPAS flight area at the north site was approximately 1.1 km by 0.5 km, and the flight area at the south site was 1.0 km by 0.7 km. The RPAS flew over the site at a speed of 8 metres per second and an altitude of 60 m above the ground (approximately 30 m above the tree canopy) in a ‘lawnmower’ pattern^42^. All RPAS surveys were conducted at first light with ambient temperatures of 8.0–21.7 °C and wind speeds of 6–17 kilometres per hour (Table 1).

**Table 1:** Time and weather conditions for RPAS surveys conducted at Petrie North and South sites.

Survey Site and Number	Survey Type	Date	Time	Ambient Temperature (°C)	Wind Speed (kilometre/hour)
North 1	Training	27/02/2018	5:25AM	21.7	17
North 2	Training	22/05/2018	5:58AM	14.2	13
North 3	Training	12/06/2018	5:47AM	10.0	12
North 4	Testing	10/07/2018	6:15AM	9.4	14
North 5	Testing	24/07/2018	5:52AM	9.0	11
North 6	Testing	07/08/2018	6:14AM	15.4	11
South 1	Training	23/05/2018	5:49AM	14.3	13
South 2	Training	13/06/2018	6:00AM	11.2	14
South 3	Testing	11/07/2018	5:51AM	10.1	12
South 4	Testing	25/07/2018	5:41AM	10.8	6
South 5	Testing	08/08/2018	5:34AM	8.0	13

### Verifying Automated Detection Output

The algorithm was then applied to the resulting thermal imagery and an output of koala detections generated using the methods described in ‘Algorithm Workflow’ above.

The GPS co-ordinates of ground-surveyed koalas and the co-ordinates of each logged detection, as well as the flight path of the RPAS were superimposed on to a map of the site terrain using GPSVisualizer^43^. The number of koalas within the RPAS survey area was then recorded.

**Figure 3:**
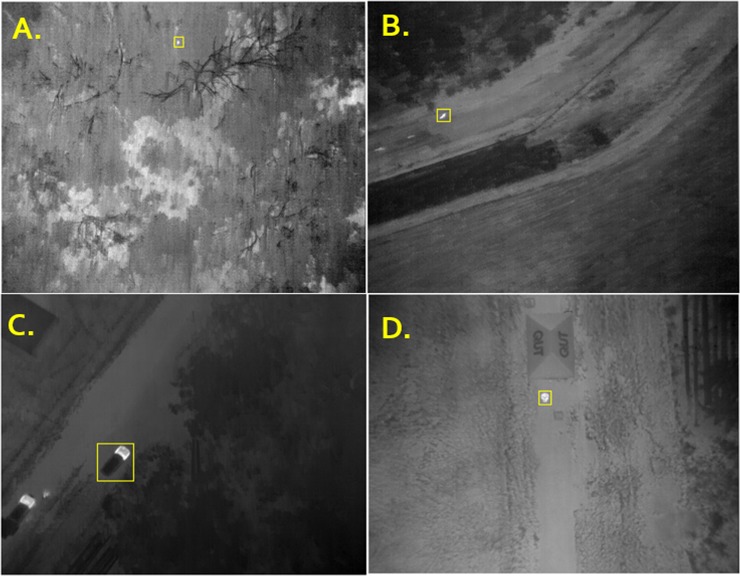
Objects automatically detected in RPAS-derived thermal imaging: (**A**) Candidate koala signature, (**B**) Kangaroo, (**C**) Car, (**D**) Human.

In ThermoViewer^44^, frames in which detections were logged were examined to determine whether a thermal signature was visible within the bounding box co-ordinates generated by the algorithm. If no thermal signature was present within the bounding box it was recorded as a false positive. If a thermal signature was detected within the bounding box the signature was classified according to the following criteria: Candidate signatures were round, located in tree canopies, 3–30 pixels in wide, and displayed no or minimal movement between frames; Kangaroo thermal signatures were located in open terrain such as grass fields, moved rapidly between frames and/or were greater than 20 pixels wide; and Car and Human thermal signatures were easily recognizable and distinguishable from other signatures (Fig. 3).

Candidate signatures with GPS co-ordinates that could be matched to the location of a ground-surveyed koala were then identified as belonging to that koala. Detected candidate signatures that did not match the location of a ground-surveyed koala, and the source of which could not be identified were then labelled as ‘other’ signatures. This category included detections where no thermal signature could be identified as well as unidentifiable signatures, the source of which may have included: uncollared immigrant koalas that could not be verified as true positives; possums; large birds; and other animals of similar size and shape to koalas. In cases where multiple detections were recorded within a single frame, visual inspection of all bounding boxes was conducted to determine if they belonged to the same or different signatures.

### Manual Detection

We also determined the accuracy of detecting koalas in RPAS-derived thermal imagery using manual assessment. This was conducted independently from the automated identification and before any results from automated detection were known. RPAS-derived thermal imagery was viewed using ThermoViewer and examined frame by frame for candidate signatures. Candidate signatures were defined using the same criteria as described for automated detection. Upon detection of a candidate signature, footage was played frame by frame and the frames in which the signature first appeared and disappeared from view were recorded, along with the GPS co-ordinates.

GPS co-ordinates of candidate signatures and ground-surveyed koalas were then mapped in GPSVisualizer. Candidate signatures were then identified as ground-surveyed koalas or labelled as ‘other’ signatures using the same methods used to match automated detections to locations of ground-surveyed koalas.

### Data Analysis

Ground-surveyed koalas that were automatically detected in thermal imaging derived from RPAS surveys were considered ‘true positives’. Given that the likelihood of detecting an uncollared koala was minimal, we were able to use the number of true positives and the total number of collared koalas present in the survey area to calculate a conservative estimate of probability of detection for automated and manual detection methods as:

$${P}_{d}=\frac{{K}_{detected}}{{K}_{obs}}$$

Where *K*_*detected*_ is the number of koalas correctly identified by the automated method, and *K*_*obs*_ is the total number of collared koalas present on site during RPAS surveying. To quantify the precision of each method, precision rate was estimated as:

$$PR=\frac{{K}_{detected}}{({K}_{obs}+{K}_{false})}$$

Where *K*_*false*_ is the number of ‘other’ signatures falsely identified or unable to be identified as koalas in each survey. Accuracy of the methods in terms of magnitude and variance in error was also compared using both root mean square error (RMSE) and mean absolute error (MAE). These measures were estimated as:

$$\begin{array}{ccc}RMSE & = & \sqrt{\frac{{\sum }_{i=1}^{n}{({K}_{obs,i}-{K}_{detected,i})}^{2}}{n}}\\ MAE & = & \frac{1}{n}\sum _{i=1}^{n}|{K}_{obs,i}-{K}_{detected,i}|\end{array}$$

Where $i$ is time/place of surveying and $n$ number of surveys.

In order to assess the efficiency of each method, the time taken to identify all possible candidate signatures automatically and manually for each survey was recorded.

## Results

As is typical for machine learning studies, both the training set and testing sets were evaluated independently. For the training datasets (North 1–3 and South 1–2), the automated method had a higher probability of detection overall (85%) than the manual method (52%), with a range of 68–100% detection compared to 40–55% detection for manual (Table 2). The overall precision of the algorithm for the training datasets (60%) was also higher than that yielded using the manual method (34%), with a range of 47–69% in each survey for automated and 29–54% for manual (Table 2).

**Table 2:** Probability of detection and precision rate for ground-surveyed koalas in RPAS-derived thermal imaging from north and south sites at Petrie, QLD by automated and manual methods.

Survey Site and Number	Koalas Detected by Survey Method						
	Ground Survey Count	Automated			Manual		
		Count	Probability of Detection (%)	Precision Rate (%)	Count	Probability of Detection %	Precision Rate (%)
North 1*	19	13	68	48	9	47	29
North 2*	20	15	75	54	11	55	50
North 3*	20	20	100	69	11	55	37
North 4	15	14	93	71	10	67	17
North 5	18	17	94	69	13	72	29
North 6	19	15	79	60	12	63	50
South 1*	5	5	100	47	2	40	29
South 2*	11	11	100	52	6	55	54
South 3	9	7	78	58	5	56	35
South 4	11	9	82	45	6	55	50
South 5	6	6	100	43	3	50	18

*Dataset included in training.

For the testing datasets, the automated detection method also yielded a higher overall probability of detection of 87%, with a range 78–100% per mission, compared to manual detection which resulted in an overall probability of detection of 63%, ranging between 50–72% per mission (Table 2). The difference in performance was greatest in surveys of the south site where the automated method gave a 78–100% probability of detection compared to 50–56% for the manual method (Table 2). At the north site, the probability of detection for the automated method (79–94%), was also higher than the manual method (63–72%) in each survey (Table 2). The overall precision rate achieved using the automated method (49%) was higher than that of the manual method (41%). At the south site, the precision rate for automated detection was 43–58% compared to 18–50% for manual detection (Table 2). At the north site, the automated method yielded a precision rate of 60–71% and the manual method was to found to have a 17–50% precision rate in each survey (Table 2).

The automated method was also found to more accurately detect koalas than the manual method in terms of both RMSE and MAE, with mean RMSE of the manual method (5.7321) being 2.97 times larger than the automated method (1.9272), and mean MAE of the manual method (5.4286) 3.80 times larger than the automated method (1.4286).

The main source of false positive errors that affected precision rate for automated detection were ‘other’ signatures, which accounted for 55% of total misidentified objects, followed by kangaroos (29%), cars (12%), and humans (4%) (Table 3). The only source of false positives for manual detection were ‘other’ thermal signatures (Table 3).

**Table 3:** Objects falsely identified as koalas in RPAS-derived thermal imagery using automated and manual methods.

Object Detected	Automated Method	Manual Method
Kangaroo	21 (29%)	0 (0%)
Car	9 (12%)	0 (0%)
Human	3 (4%)	0 (0%)
Other	40 (55%)	51 (100.0%)
**Total**	**64 (100%)**	**41 (100.0%)**

The mean time taken to automatically detect koalas in thermal footage from one RPAS survey was 136 minutes, which was shorter than the mean time of 170 minutes taken to complete manual detection. Note however that a large portion of the 136 minutes for the automated detection is processing time, with no input or attention from the operator needed.

## Discussion

The results of this study demonstrate that combining published object detection algorithms with RPAS-derived thermal imagery can be used as an accurate and precise method for the detection of koalas in complex environments. The probability of detection found in this study (78–100%) was higher than that which was found in previous studies that used automated methods to detect other threatened, low-density species such as dugongs (48.6–51.6%) and species in complex environments such as bears (60%) in aerial imaging^1,25^. Surprisingly, it was also performed as well or better than the 43–100% probability of detection rate found in various studies in which species that occur in high aggregations (nesting seabirds), open environments (large African mammals), or semi-natural environments (captive deer) were surveyed^23,24,26–32^. The fact that the current method achieved these high rates of detection in a highly structurally complex environment compared to other studies provides strong support for the utility of our automated detection method, although further evaluation with more data and using different species is warranted to evaluate the robustness of the methodology. In this study we have shown that the probability of detection using this method was comparable to previous studies despite the koalas surveyed in this study being sparsely distributed, in natural environments, over a larger area and featuring more complex canopy cover than the simpler and more artificial settings surveyed in prior research^23,24,26–31,45^. There is a clear route for application of the method for conservation, even at this stage of development. While the automated method does result in a number of false alarms, for application in new areas it is easy to quickly review the detection outputs upon completion and discard false detections. Obvious false alarms such as those caused by people, vehicles and livestock can be quickly discarded with minimal effort.

In all testing RPAS surveys conducted in this study, the probability of detection yielded by automated detection also exceeded direct observational surveys (60–75%)^21,22^. This suggests the current methodology is a promising way forward for koala monitoring, as it has been shown to be more sensitive than the most accurate method commonly used for the species even with minimal algorithmic training, in addition to being less time-consuming and reliant on expert observers^19,21^.

Automated detection was also found to be more accurate than manual examination of the RPAS-derived images in terms of probability of detection, RMSE and MAE. This is consistent with previous findings that manual assessment of RPAS-derived imaging is more prone to false negative error than semi-automated and automated detection methods, leading to a greater average magnitude of error between predicted and actual population size^13,14^.

Automated detection was demonstrated to be more precise than manual detection which was consistent with predictions from previous studies^13,14^. The overall precision rate of 49% suggests the number of koalas on site was overreported by the algorithm. This is apparently the first study to report precision rates with a method using direct identification of individual koalas however, and so it is unknown if this presents an improvement on detections in the field using direct koala observation. Nonetheless, it was equivalent to the upper range of performance reported in previous attempts to automatically detect wildlife in RPAS-derived imaging (7–67%)^23–29^. We note also the trade-off between precision and probability of detection. Algorithmic approaches to remotely-sensed detection of wildlife with a high probability of detection typically yield a very low precision rate. Two previous algorithms for detecting large mammals in the African savannah yielded a 10–40% precision rate with 75–80% probability of detection, less than the 87% probability of detection reported in this study despite the higher relative complexity of habitat surveyed in the current study^23,25^.

It is also important to note that the precision rates reported in this study are conservative estimates as it was assumed all candidate signatures that were not able to be matched to collared koalas were false positives rather than true detections of uncollared koalas. The immigration rate has been estimated by field ecologists to be a maximum of 10%, and if this were factored into the estimate, precision could potentially increase up to 63–100% in each mission^22^.

Although the current study already presents strong evidence for the utility of the method, collection of additional data and further training of the algorithm using annotated images of known koala and non-koala heat signatures gathered during RPAS-surveys will further improve the precision of the automated method for detecting koalas described in this study. Future work including adjustments to algorithm sensitivity, improved tracking of detected objects and parameters for scale of target objects should further reduce spurious detections. Nonetheless, even in its current form this methodology has already matched the optimal of performance of similar wildlife automated detection methods.

Automated and manual detection methods were found to be similarly efficient in terms of time required to complete detections per survey, although as noted much of the time required for the automated detection method is simply processing time and requires no input from the user. Automated detection yields a higher probability of detection and a lower or comparable rate of false positive error in the same amount of time as the latter.

Overall, the automated method described in this study has been shown to be a reliable tool for detecting koalas in RPAS-derived thermal imagery with the capacity for immediate application. It provides information regarding the presence and abundance of koalas that is more efficient, non-invasive, and which requires less survey effort than current commonly-used methods such as on-ground observational surveys. It also requires comparable survey-effort in terms of time to manual assessment of thermal imagery^19,21^. There is a wide range of potential applications for the information obtained using this automated detection method, including informing decisions about the conservation, management, and policy regarding koalas, and monitoring the impact of interventions on population size and trends. With further training and refinement to increase accuracy and reduce false or redundant detections, this method could also be incorporated into methods of abundance estimation and predict trends in population size.

There is also potential for the algorithm to be trained to detect other mammal species that are low-density, cryptic, sensitive to human disturbance, or inaccessible. In particular, this approach could be applied to remotely survey species in environments that are more complex and have greater visual obstruction to viewing animals than previous methods were limited to^1,23^. This automated method could be used to detect the presence of and monitor the abundance of both of species of conservation concern, as well as invasive species. It is recommended that further RPAS-surveys using thermal imaging be undertaken to collect more training and testing data to improve the accuracy, precision and efficiency of both the algorithm and survey design for detecting koalas as well as other species, and ensure maximum potential benefits of this new method to researchers, conservation managers and policy makers are realized.

## Data Availability

The datasets collected and analysed in this study including RPAS-derived thermal imagery is available from the corresponding author on reasonable request.
